# *In vitro* histological investigation of interactions between rat decellularized large intestine scaffold and human adipose derived mesenchymal stem cells

**Published:** 2015-09-15

**Authors:** Somayeh Naderi, Malihe Akbarzadeh Niaki, Nasser Mahdavi Shahri, Maryam Moghaddam Matin, Masoud Fereidoni, Fatemeh Naseri

**Affiliations:** 1 *Department of Biology, Faculty of Science, Ferdowsi University of Mashhad, Mashhad, Iran; *; 2 *Stem Cell Biotechnology Research Group, Institute of Biotechnology, Ferdowsi University of Mashhad, Mashhad, Iran; *; 3 *Cell and Molecular Biotechnology Research Group, Institute of Biotechnology, Ferdowsi University of Mashhad, Mashhad, Iran; *; 4 *Stem Cell and Regenerative Medicine Research Group, Iranian Academic Center for Education, Culture and Research (ACECR),Khorasan Razavi Branch,Mashhad, Iran; *; 5 *Central Laboratory, Ferdowsi University of Mashhad, Mashhad, Iran.*

**Keywords:** Extracellular matrix, Large Intestine, Mesenchymal stem cells, Rat, Scaffold

## Abstract

The aim of this study was to investigate the interactions between rat intestine decellularized scaffold and human adipose derived mesenchymal stem cells. Rat large intestine was dissected in fragments and decellularized by physicochemical methods. The scaffolds were loaded by human adipose derived mesenchymal stem cells expressing green fluorescent protein. Microscopic sections were prepared from the scaffolds after two weeks of culture with stem cells and studied by histological methods. The interactions of scaffolds with MSCs were also studied by electron microscopy. Histological and electron microscopy studies revealed human mesenchymal stem cell adhesion, migration, division and maintenance during the 14 days of culture *in vitro*. According to the results, scaffolds prepared from rat intestine matrix could be a suitable scaffold for studying *in vitro* cell behaviors such as division, migration and attachment. These various behaviors of cultured cells might be due to inductive effects of the extracellular matrix derived scaffold. However, more investigations are required to discover the exact effects of this scaffold and its interactions with mesenchymal stem cells.

## Introduction

Cells are able to interact with their surrounding extracellular matrix (ECM), which regulates the cellular behaviors by affecting on morphology, survival, proliferation, migration and differentiation of the cells. The scaffold acts as a template for cell organization and tissue development in tissue engineering process. Biologic scaffolds prepared from ECM of decellularized mammalian tissues have been shown to facilitate constructive remodeling in injured tissues.^1^^,^^2^ Decellularization is an important process to prepare an ECM derived scaffold. Many methods including physical, chemical and enzymatic protocols have been developed to reach the goal of decellularization.^2^ A wide range of decellularized tissues, which retain both structure and molecules of the ECM have been studied for tissue engineering and regenerative medicine applications.^3^^-^^8^ The ECM is a molecular complex composed of molecules like collagen, elastin, glycoproteins, proteoglycans, glycosaminoglycans and proteins like growth factors, cytokines, enzymes and their inhibitors and plays role in various processes like cell adhesion, growth, migration and differentiation.^9^^-^^12^

We have focused on using human adipose derived mesenchymal stem cells (AD-MSCs) because they are an attractive and readily available source of adult stem cells, which due to ease of harvest, abundance and their immunomodulating properties are popular for use in many stem cell applications. They have been widely studied as an autologous mesenchymal cell source to produce chondrocytes, osteoblasts, and fibroblasts.^12^^-^^16^ This study was aimed to develop a decellularized rat large intestine scaffold using a combination of physical and chemical methods and then investigate the inductive effects of this scaffold on seeded AD-MSCs.

## Materials and Methods

**Decellularization process.** In this experimental research, adult male Wistar rats (n = 4) weighting 250 - 300 g were used. Animal experiments were performed according to the Iranian Council for the Use and Care of Animals Guidelines and were approved by the Animal Research Ethical Committee of Ferdowsi University of Mashhad, Mashhad, Iran. After removing the large intestine from male Wistar rats, it was washed with normal saline, and dissected in cylindrical shapes with 7 mm height. Intestine pieces were washed with sterile phosphate-buffered saline (PBS), immersed in liquid nitrogen (− 196 ˚C) for 2 min and thawed in distilled water and then PBS at room temperature for 5 min. The freeze/thaw process, which leads to cell lysis, was repeated five times. In the chemical phase of decellularization, specimens were treated with 1% (*w/v*) sodium dodecyl sulfate (SDS) solution (Merck, Darmstadt, Germany) for 24 hr at 37 ˚C. 

Then, in order to reduce residual SDS from the scaffolds and to sterilize them, two washing steps were performed. Hence, specimens were washed first with 70% ethanol to remove residual SDS from tissues and a second wash with PBS was performed for 60 min at room temperature to complete the decellularization process.^17^

**Cell seeding and culture method.** The scaffolds were cultivated with the AD-MSCs expressing green fluorescent protein (GFP).^18^ In this regard, after sterilization, decellularized scaffolds were transferred to 12-well plates and seeded with 100 μL aliquots containing 5 × 10^5^ cells and incubated at 37 ˚C with 5% CO_2_ in air for 1 hr to allow cell attachment. In the final step, seeded scaffolds were immersed in 2 mL Dulbecco’s modiﬁed Eagle’s medium (Gibco, Paisley, Scotland) supplemented with 15% fetal bovine serum (Gibco) and 100 µL penicillin/streptomycin (Biosera, Sussex, UK), which was changed every two day. Unseeded scaffolds were used as controls, and all samples were subjected to histological staining and scanning electron microscopy on day 14 after cell seeding.

**Histological studies.** All samples were fixed in 4% paraformaldehyde solution and dehydrated through a graded series of ethanol, embedded in paraffin, cross-sectioned at a thickness of 5 μm with a microtome (Leits, Vienna, Austria), deparaffinized by xylene, rehydrated, and stained appropriately. To determine construct cellularity, hematoxylin and eosin (H & E) staining was used. To detect the labeled AD-MSCs, the sections were deparaffinized and observed by a fluorescent microscope.

**Scanning electron microscopy (SEM).** Scanning electron micrographs were taken to examine the surface topology of prepared scaffolds. In order to prepare samples for electron microscopy, specimens were fixed with 2.5% gluteraldehyde for 24 hr at room temperature and then washed with PBS for three times. Then, they were dehydrated in an ethanol-graded series (20%, 50%, 70%, 90%, 100%). Subsequently, the samples were examined under a scanning electron microscope (Leo VP 1450; Carl-Zeiss, Oberkochen, Germany) after coating with gold.

**Transmission electron microscopy (TEM).** For TEM evaluation, specimens were fixed with gluteraldehyde and 1% osmium tetroxide and dehydrated with incremental concentrations of ethanol. Specimens were placed in propylene oxide for 30 min, and finally placed in pure resin for 30 min (Araldite 502 resin kit; TAAB, Aldermaston, UK). The specimens were segmented into a thickness of 80 nm with ultra-microtome (LKB, Bromma, Sweden). These sections were studied and photographed with a TEM (Leo 910; Carl-Zeiss, Oberkochen, Germany).

## Results

**Fig. 1:**
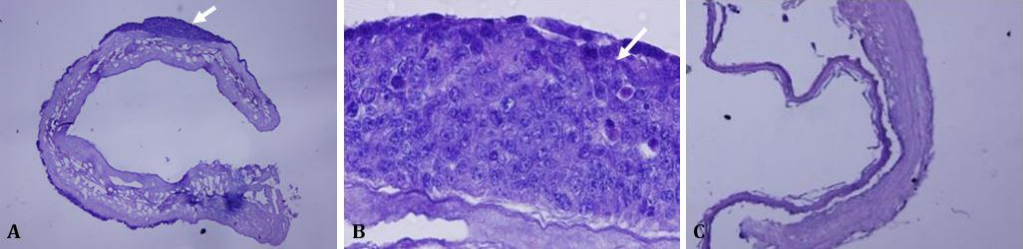
Transverse sections prepared from large intestine scaffold. **A **and** B)** after culture with AD-MSCs (arrows); **C)** control scaffold  (H & E, A and C 40×; B 400×). AD-MSCs were spread on scaffold surface and migration took place inside the matrix. The morphology and attachment of seeded cells on the scaffold are illustrated

A combination of physical and chemical decellularization methods was used in this research. Snap freeze-thaw using liquid nitrogen as a physical method and 24 hr treatment with 1% SDS as chemical method, resulted in elimination of cells while preserving intestine structure. AD-MSCs were seeded on the scaffolds for up to two weeks. On day 14, AD-MSCs were spread on scaffold surface and migration took place inside the matrix. The morphology and attachment of seeded cells on the scaffold are illustrated in Figure 1. The AD-MSCs were able to form epithelium-like structures at two weeks after culture.

**Fig. 2:**
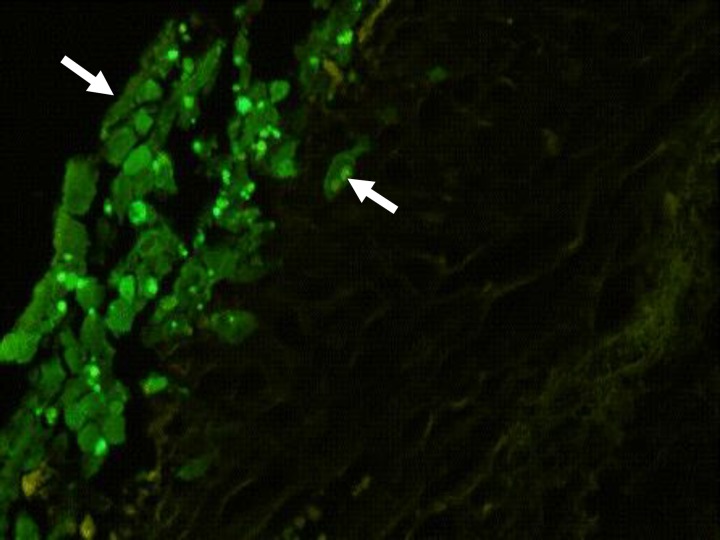
Transverse sections prepared from large intestine scaffold. GFP labeled AD-MSCs (arrows) are attached on the surface of the scaffold (200×).

**Fig. 3:**
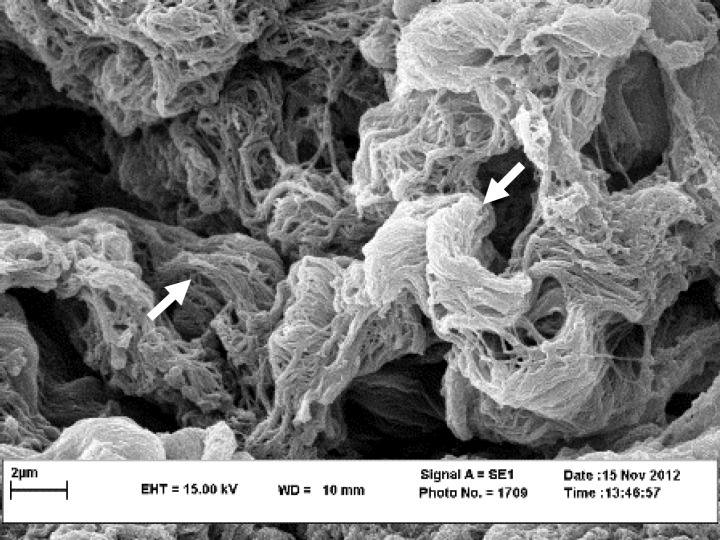
SEM images of the decellularized large intestine tissue. SEM of scaffold demonstrated the collagen fibers (arrows) in decellularized scaffold.

**Fig. 4:**
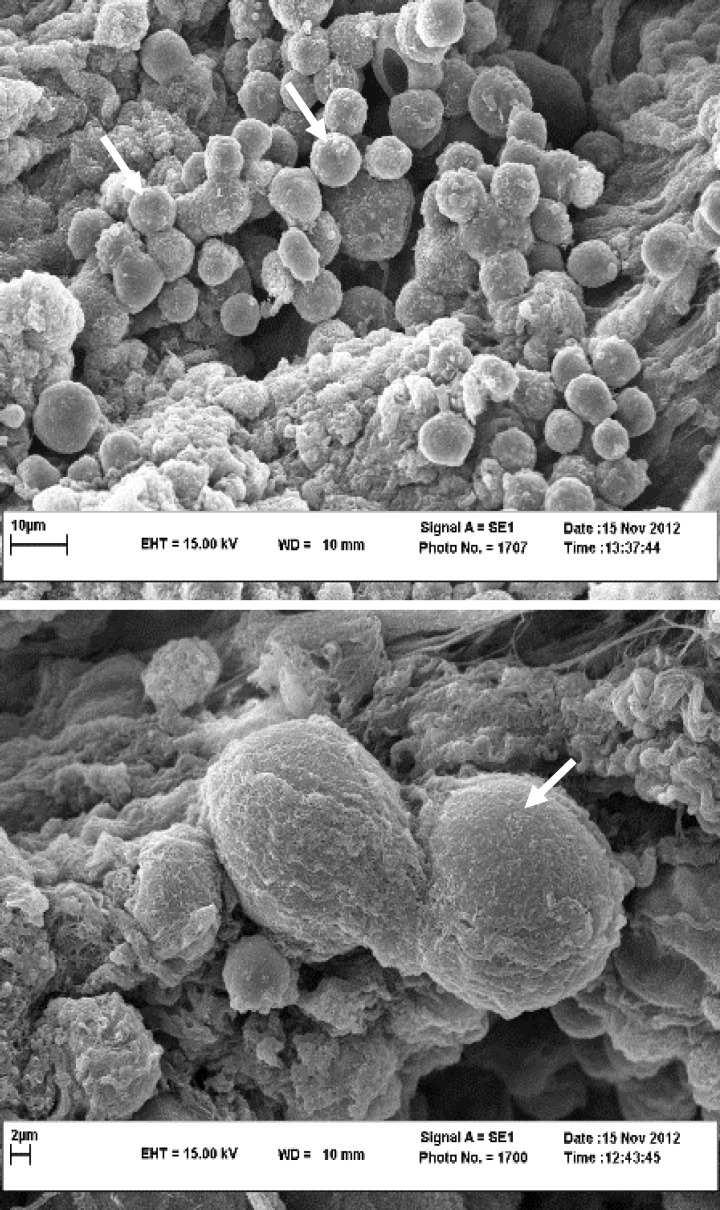
**A, B)** SEM images of the decellularized large intestine scaffold after two weeks of culture with AD-MSCs. Cell attachments are obvious on the surface of the scaffold (arrows

**Fig. 5:**
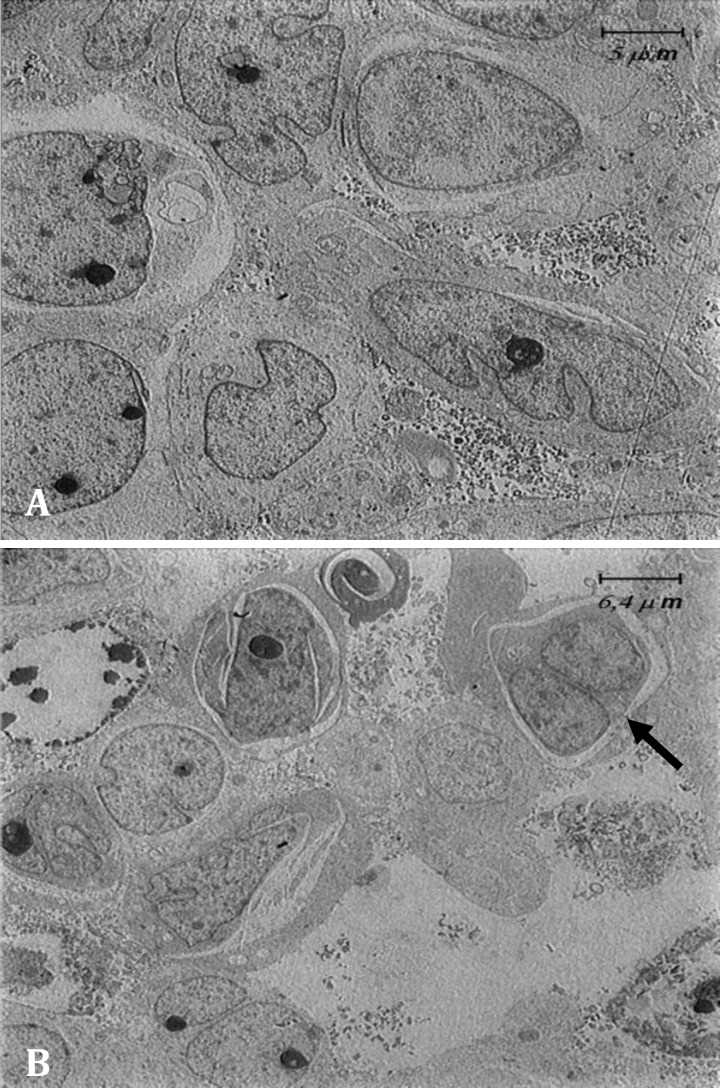
TEM image of the decellularized large intestine scaffold after two weeks of culture with AD-MSCs. **A, B)** AD-MSCs are obvious in the scaffold. Cell division is indicated by arrow

Penetration and survival of GFP labeled AD-MSCs were detected, two weeks after culture (Fig. 2). The SEM micrographs revealed preservation of collagen and elastin fibers in decellularized scaffolds (Fig. 3). The SEM micrographs of seeded scaffolds also demonstrated the adherence and attachment of AD-MSCs to scaffolds two weeks after culture (Fig. 4). The TEM micrographs revealed cells and also cell division after initial seeding (Fig. 5).

## Discussion

The aim of this study was to investigate the inter-actions between rat decellularized intestine scaffold with human AD-MSCs. Tissue engineering systems are being used as model systems to study cellular behaviors. It is essential to understand the mechanisms involved in interaction of cells with their ECM. The cells of each tissue are able to interact with their surrounding (i.e. ECM), which regulates the cell behaviors by affecting on morphology, survival, proliferation, migration and differentiation of the cells. Several researches have focused on interactions between various cell types and a wide range of synthetic or natural scaffolds to find pieces of the puzzle in tissue engineering. Several studies have been performed to demonstrate the inductive effects of different ECM scaffolds on behaviors of seeded cells. The chemical composition and physical properties of natural ECM have been shown to prominently influence cell morphology, motility, and migration. Cell-ECM interactions are accompanied by cytoskeletal action, matrix remodeling and contraction, which modulate cell fate.^19^^-^^24^ Furthermore, the relationship between contractile forces, resulting in tensile stresses in the cytoskeleton forces and the mechanical stiffness, or elasticity of the ECM can have major influence on cell behaviors such as migration, apoptosis, and proliferation.^25^^-^^27^

Beckstead *et al*. compared esophageal epithelial cell interactions with Allo-Derm, the decellularized skin product, and degradable polyesters, and demonstrated that esophageal epithelial cell adhesion and proliferation were supported by both natural and synthetic scaffolds, however, the natural scaffold showed superior morphology compared to the synthetic scaffold.^28^ Rodrigues *et al*. showed that fibroblast seeding onto acellular dermal matrix for 14 days can allow good conditions for cell adhesion and spreading on the matrix, whereas, migration inside the matrix was limited.^29^ In the present study, although cells adhered and spread on decellularized intestine scaffold surface, the low number of cells inside the matrix, suggested that during the culture interval cells were unable to alter the dense organization of the collagen bundles, which acted as a physical barrier. The SEM and TEM images proved successful adhesion and proliferation of cells on the scaffold at 14 days after culture and indicated the microstructure of decellularized scaffolds with a suitable interconnected structure which facilitated cell adhesion and the transportation of nutrients and waste during cell culture. Findings of the present study showed that cell division was detectable in penetrated AD-MSCs in to the scaffold after two weeks of culture. These various behaviors of cultured cells might be due to inductive effects of the large intestine ECM derived scaffold. Several studies have been performed to demonstrate the inductive effects of different ECM scaffolds on behaviors of seeded cells. For instance, Lindberg and Badylak demonstrated the ability of small intestinal submucosa ECM to support epidermal cell and fibroblast attachment, migration, proliferation and differentiation with deposition of basement membrane components.^26^ Ozeki *et al*. seeded esophageal epithelial cells inside decellularized esophagus and demonstrated the potential of the ECM derived scaffold to induce the polarity, proliferation and differentiation of seeded cells.^30^ In agreement with these studies, the results of the present study demonstrated retaining of main ECM components such as collagen and elastin after decellularization, and also showed migration and proliferation of cultured AD-MSCs. It could be concluded that decellularized scaffold utilized in this study may have induced the adhesion, migration and proliferation of cultured AD-MSCs. More experiments to detect the differentiation of penetrated cells into the scaffold are required to improve our knowledge about cell – matrix interactions.
